# Associations between neuropsychiatric symptoms and incident Alzheimer’s dementia in men versus women

**DOI:** 10.1007/s00415-022-11541-w

**Published:** 2022-12-26

**Authors:** Ioannis Liampas, Vasileios Siokas, Constantine G. Lyketsos, Efthimios Dardiotis

**Affiliations:** 1 Department of Neurology, Faculty of Medicine, School of Medicine, University Hospital of Larissa, University of Thessaly, Mezourlo Hill, 41100 Larissa, Greece; 2 Department of Psychiatry and Behavioral Sciences, Johns Hopkins School of Medicine, Baltimore, MD 21205 USA

**Keywords:** Sex differences, Depression, Apathy, Agitation

## Abstract

**Objective:**

To examine whether associations between individual neuropsychiatric symptoms (NPS) and incident Alzheimer’s dementia (AD) differ in men versus women.

**Methods:**

Data were acquired from the National Alzheimer’s Coordinating Center (NACC) Uniform Data Set. Two sets of older (≥ 60 years) participants were formed: one of cognitively unimpaired (CU) individuals, and one of participants with mild cognitive impairment (MCI). NPS were assessed using the Neuropsychiatric Inventory Questionnaire. Cox proportional hazards models examined associations between individual NPS and AD incidence separately for each participant set. These models featured individual NPS, sex, NPS by sex interactions as well as a number of covariates.

**Results:**

The analysis involved 9,854 CU individuals followed for 5.5 ± 3.8 years and 6,369 participants with MCI followed for 3.8 ± 3.0 years. NPS were comparably associated with future AD in men and women with MCI. Regarding CU participants, the following significant sex by NPS interactions were noted: female sex moderated the risk conferred by moderate/severe apathy (HR = 7.36, 3.25–16.64) by 74%, mitigated the risk conferred by moderate/severe depression (HR = 3.61, 2.08–6.28) by 52%, and augmented the risks conferred by mild depression (HR = 1.00, 0.60–1.68) and agitation (HR = 0.81, 0.40–1.64) by 83% and 243%, respectively.

**Conclusions:**

Apathy, depression and agitation were differentially associated with incident AD in CU men and women. No individual NPS was associated with different risks of future AD in men versus women with MCI.

**Supplementary Information:**

The online version contains supplementary material available at 10.1007/s00415-022-11541-w.

## Introduction

Neuropsychiatric symptoms (NPS) are almost universal among older adults with mild cognitive impairment (MCI) and Alzheimer’s dementia (AD) [1, 2]. In cognitively unimpaired (CU) individuals, NPS have been linked to more precipitous cognitive decline [3] and elevated risk of incident MCI or AD [4, 5]. Further, NPS have been associated with a greater risk of progression from MCI to AD [6] and steeper cognitive trajectories in patients with AD [7]. Therefore, regardless of the exact underlying pathophysiological connection, the presence of NPS in older adults is a predictor of worse cognitive impairment and AD.

Biological sex appears to modify the course of AD. Different sex-related associations have been reported for prognosis, risk factors and drug effects (to name a few aspects of sex interactions) [8]. However, sex effects have received limited attention in NPS research. Some have reported substantial differences in NPS between men and women: women present with a broader range of NPS and carry a greater neuropsychiatric burden compared to men [9]. Further, psychotic and affective symptoms afflict women more frequently, whereas apathy appears to be more prevalent among men with AD [10].

Despite the evidence of sex-specific NPS patterns in AD, the predictive value of NPS has not been explored separately in men and women. Although psychotic and affective symptoms, apathy, agitation, irritability, and aberrant motor behaviour have been related to faster progression from normal aging to MCI and ultimately AD, it is not clear whether these associations differ between men and women [5, 11–14]. The aim of the present study was to examine the predictive properties of NPS separately in male and female older adults. We capitalised on longitudinal data from the Uniform Data Set (UDS), a set of prospectively collected data on volunteers from multiple Alzheimer's disease research centres (ADRCs) across the United States.

## Methods

UDS is a central repository of collaborative-multidisciplinary data collected in National Institute on Aging/NIH—funded ADRCs across the United States stewarded by the National Alzheimer's Coordinating Center (NACC). The key methodological features of the UDS have been detailed elsewhere [15–17]. In brief, it was initiated in 2005 and operates to date, as a resource for dementia research. Participants are recruited according to each ADRC’s distinct protocol and undergo standardized evaluations on an approximately annual basis. Participants or surrogates provide informed consent before participation. All procedures are overseen by Institutional Review Boards at each ADRC and have therefore been performed in accordance with the ethical standards laid down in the 1964 Declaration of Helsinki and its later amendments.

### Eligibility criteria and participant selection

The current study was based on data from the subset of older (≥ 60 years) NACC participants enrolled between September 2005 (inception of the UDS) and December 2021 (data freeze) from a total of 43 ADRCs. Two distinct groups of individuals were assembled. The first involved every individual who was CU at entry to the NACC (CU set). The second included individuals diagnosed with MCI at entry to the NACC plus those who developed MCI at follow-up, without a prior dementia diagnosis (MCI set). For those who developed MCI at follow-up, the first visit at which they were diagnosed with MCI was the starting point of their monitoring. Participants with a follow-up diagnosis of dementia other than AD were excluded (to avoid competing risks of conversion to non-AD dementias).

Cognitive diagnoses were established by either an interdisciplinary consensus team (in the majority of cases) or a single clinician (who conducted the examination), depending on the specific requirements of each ADRC’s protocol. Diagnoses were based on medical history, neuropsychological performance, and psychosocial functioning. CU was defined by the absence of a diagnosis of dementia, MCI or cognitive impairment not MCI, according to the physician-based diagnosis. MCI and dementia were diagnosed using standard clinical criteria [18–23]. Participants with cognitive impairment who did not clearly fit into the categories of CU, MCI, or dementia were diagnosed as cognitively impaired, not MCI.

Individuals reporting treatment with an FDA-approved medication for AD (i.e. tacrine, donepezil, rivastigmine, galantamine and memantine) were excluded from both sets (receiving such medication raised doubts about the credibility of the clinician-based diagnoses). Participants with a physician’s diagnosis of a psychiatric disorder (schizophrenia, bipolar disorder, post-traumatic stress disorder, obsessive–compulsive disorder and developmental neuropsychiatric conditions) were also excluded from both groups to eliminate the confounding of long-standing neuropsychiatric manifestations that interfere significantly with cognition.

### Measurement of NPS

The Neuropsychiatric Inventory Questionnaire (NPI-Q) is an informant administered, widely used tool for the evaluation of NPS in dementia research [24]. NPI-Q evaluates 12 domains: delusions, hallucinations, agitation/aggression, depression/dysphoria, anxiety, elation/euphoria, apathy/indifference, disinhibition, irritability/lability, aberrant motor behaviour, night-time behaviours, and eating behaviours. Informants initially report the presence or absence of cardinal symptomatology for each domain in the month preceding the examination and subsequently rate the severity of any symptoms according to a 3-point severity scale: mild (noticeable, but not a significant change); moderate (significant, but not a dramatic change); or severe (very marked or prominent; a dramatic change). For most NPI-Q domains, participants were grouped according to NPS on a 3-point scale: 0: absent; 1: mild; 2: moderate and severe symptomatology (e.g., irritability: absent, mild, moderate/severe; anxiety: absent, mild, moderate/severe and so on). For the domains of delusions, hallucinations, elation/euphoria and aberrant motor behaviour, owing to the very small number of participants with moderate/severe symptomatology, participants were dichotomized for presence of these NPS (0: absent; 1: mild, moderate and severe symptomatology) [25].

### Factors and covariates considered

Age at the time of the baseline evaluation and education in years of formal schooling were treated as scale variables. Sex, race (Caucasian, African American, American Indian or Alaska Native, Native Hawaiian or Pacific Islander, Asian and multiracial), number of apoE4 alleles (0 or 1 or 2), as well as a number of comorbidities, medications and habits that may confound the relationship between NPS and cognitive decline were treated as categorical variables: history of seizures, traumatic brain injury (TBI), Parkinson’s disease (PD), cerebrovascular disease (CEVD), cardiovascular disease (CAVD), diabetes mellitus (DM), hypertension, dyslipidaemia, smoking history, alcohol abuse or other substance abuse (with clinically significant impairment occurring over a 12-month period manifested in one of the following areas: work, driving, legal, or social), vitamin B12 deficiency, reported use of antidepressants, reported use of antipsychotics and reported use of anxiolytic/sedative/hypnotic agents. These parameters were positively assessed according to subject or co-participant reporting. To avoid over-adjustment, a statistical criterion was set for the inclusion of each factor in our analysis: only covariates that significantly differed between those without and those with at least one NPS were included (age, education, sex and race were accounted for regardless of the statistical prerequisite). Therefore, different sets of covariates were considered for the CU and MCI sets.

### Statistical analysis

Baseline differences between those who did and those who did not progress to AD were analysed using independent sample *t*-tests (scale variables) and Pearson’s chi-squared tests (categorical variables). Results are provided separately for the CU and MCI sets.Please confirm the section headings are correctly identified.I confirm that the headings are correctly identified

Associations between NPS and incident AD were examined using multivariable adjusted Cox proportional hazards models. Twelve separate, independent analyses were performed for each participants set, one analysis per NPS. Participants were censored at their last visit. The proportionality of hazards for different strata over time was confirmed for each model using cox regression analyses with time dependent covariates. For example, to test the proportionality of hazards for anxiety, an extended Cox model including the term anxiety*time along with anxiety was analysed. To verify that the proportionality of hazards assumption was not violated, the coefficient of the time interaction product had to be statistically insignificant.

Regarding the CU set, all analyses featured the following covariates (based on the aforementioned statistical criterion): age, education, race, PD, CEVD, CAVD, DM, hypertension, dyslipidaemia, smoking history, alcohol or other substance abuse, B12 deficiency, reported use of antidepressants, antipsychotics, or anxiolytic/sedative/hypnotic agents and sex, along with one NPS at a time (e.g., anxiety) including sex by NPS interaction terms (e.g., sex*anxiety). In the case of MCI, all 12 survival analyses featured the following covariates (based on the statistical prerequisite): age, education, race, PD, TBI, number of apoE4 alleles, alcohol or other substance abuse, B12 deficiency, reported use of antidepressants, antipsychotics or anxiolytic/sedative/hypnotic agents and sex, along with one NPS at a time (e.g., anxiety) including sex by NPS interaction terms (e.g., sex*anxiety).

To ascertain the validity of our findings, confirmatory analyses were conducted accounting for the potential confounding of preclinical cognitive alterations [26]: analyses were repeated after adjusting for baseline mini-mental state examination (MMSE) scores, as a measure of global cognition [27, 28]. To limit the amount of missing data, results from the NACC neuropsychological battery crosswalk study were utilised [29]. In specific, Montreal Cognitive Assessment (MoCA) scores were converted to equivalent MMSE scores according to the detailed conversion tables provided by the crosswalk study investigators (although the first two versions of the UDS assessed global cognition using MMSE, the more recent, third version of the UDS replaced MMSE with MoCA) [30, 31].

Statistical analyses were performed using the IBM SPSS Statistics Software Version 27 (Chicago, IL, USA). Despite performing multiple comparisons, the conventional threshold of α = 0.05 was implemented for the revelation of statistical significance. This decision was made to retain a fair statistical power for our analyses, because the great number of factors and covariates featured in our models along with the low frequency of several investigated exposures-NPS strata (e.g., delusions, hallucinations, moderate-severe depression, disinhibition, and so on) considerably undermined precision estimates (there was a risk of failing to reveal valid associations).

## Results

### CU participant characteristics and missing data

**Fig. 1:**
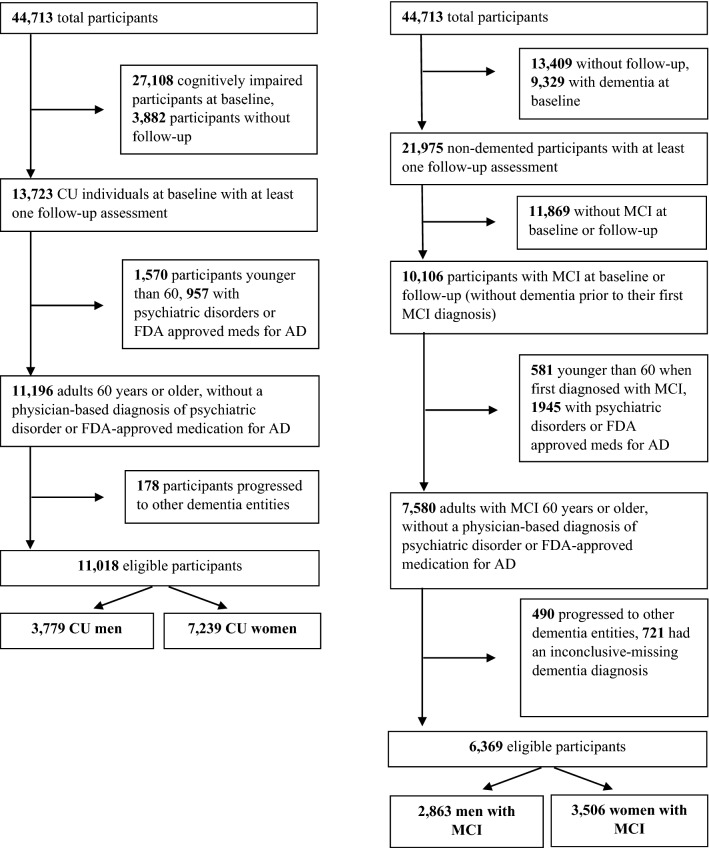
Flowcharts of participants selection

Of the 44,713 participants in UDS, 11,018 CU at baseline were eligible for the analysis (Fig. 1). A total of 621 were not included due to missing data on covariates. An additional 543 were not included in any model because of missing data on all NPS. Of the remaining 9854, between 0 and 11 individuals were excluded from each survival analysis (due to missing data on the specifically analysed NPS), with the exception of night-time behaviours (68 cases with missing data were excluded).

CU individuals with missing data (n = 1,164) were older (74.5 ± 8.5 vs. 73.0 ± 7.6 years), less educated (15.4 ± 3.4 vs. 15.9 ± 2.9 years) and more often African American or Asian compared to those without missing data (who were more often white). CEVD, DM, hypertension, night-time behaviours, depression, anxiety and irritability were more prevalent among those with missing data, while the use of antidepressants was less common (missing data analysis not shown).

Throughout the average follow-up of 5.5 ± 3.8 years (range 0.4–15.9 years), 643 older CU adults progressed to AD while 9211 did not. Baseline differences between those who did and did not develop AD are in Table 1.

**Table 1:** Baseline differences between cognitively unimpaired individuals who did and did not develop Alzheimer’s disease (AD) at follow-up

Variable	Non-demented at follow-up (*n* = 9211)	AD at follow-up (*n* = 643)	*p*-value
Age in years	72.7 ± 7.5	78.4 ± 7.3	** < 0.001**
Formal education in years	15.9 ± 2.9	15.2 ± 2.9	** < 0.001**
Sex (mela/female)	3183/6028 (34.6/65.4%)	213/430 (33.1/66.9%)	0.461
Race (Caucasian/African American/American Indian or Alaska Native/Native Hawaiian or Pacific Islander/Asian/Multiracial)	7298/1372/36/6/232/267 (79.2/14.9/0.4/0.1/2.5/2.9%)		**0.001**
Number of ApoE4 alleles (0/1/2)	5733/2122/186 (71.3/24.6/2.3%)	359/218/33 (58.9/35.7/5.4%)	** < 0.001**
Parkinson’s disease (No/Yes)	9053/158 (98.3/1.7%)	638/5 (99.2/0.8%)	0.071
Traumatic brain injury (No/Yes)	8257/907 (90.1/9.9%)	578/59 (90.7/9.3%)	0.603
History of seizures (No/Yes)	9065/134 (98.5/1.5%)	627/13 (98.0/2.0%)	0.247
B12 deficiency	8813/398 (95.7/4.3%)	617/26 (96.0/4.0%)	0.738
Alcohol abuse (No/Yes)	8964/247 (97.3/2.7%)	629/14 (97.8/2.2%)	0.441
Other substance abuse (No/Yes)	9136/75 (99.2/0.8%)	642/1 (99.8/0.2%)	0.065
Smoking history (No/Yes)	5044/4167 (54.8/45.2%)	363/280 (56.5/43.5%)	0.404
Cardiovascular disease (No/Yes)	8259/952 (89.7/10.3%)	548/95 (85.2/14.8%)	** < 0.001**
Cerebrovascular disease (No/Yes)	8727/484 (94.7/5.3%)	581/62 (90.4/9.6%)	** < 0.001**
Diabetes Mellitus (No/Yes)	8068/1143 (87.6/12.4%)	580/63 (90.2/9.8%)	0.051
Hypertension (No/Yes)	4625/4586 (50.2/49.8%)	294/349 (45.7/54.3%)	**0.028**
Dyslipidaemia (No/Yes)	4171/5040 (45.3/54.7%)	320/323 (49.8/50.2%)	**0.027**
Antidepressants (No/Yes)	7681/1530 (83.4/16.6%)	529/114 (82.3/17.7%)	0.462
Antipsychotics (No/Yes)	9172/39 (99.6/0.4%)	640/3 (99.5/0.5%)	0.871
Anxiolytics/sedatives/hypnotics (No/Yes)	8214/997 (89.2/10.8%)	589/54 (91.6/8.4%)	0.054
Delusions (No/Yes)	9176/34 (99.6/0.4%)	635/8 (98.8/1.2%)	**0.001**
Hallucinations (No/Yes)	9195/16 (99.8/0.2%)	642/1 (99.8/0.2%)	0.914
Depression (No/Mild/Moderate-severe)	8210/771/224 (89.2/8.4/2.4%)	541/72/30 (84.1/11.2/4.7%)	** < 0.001**
Anxiety (No/Mild/Moderate-severe)	8528/512/169 (92.6/5.6/1.8%)	578/49/16 (89.9/7.6/2.5%)	**0.042**
Agitation (No/Mild/Moderate-severe)	8818/302/90 (95.7/3.3/1.0%)	595/32/16 (92.5/5.0/2.5%)	** < 0.001**
Disinhibition (No/Mild/Moderate-severe)	9048/111/51 (98.2/1.2/0.6%)	620/14/9 (96.4/2.2/1.4%)	**0.003**
Irritability (No/Mild/Moderate-severe)	8312/713/182 (90.3/7.7/2.0%)	557/57/29 (86.6/8.9/4.5%)	** < 0.001**
Elation (No/Yes)	9153/56 (99.4/0.6%)	636/7 (98.9/1.1%)	0.139
Motor symptoms (No/Yes)	9117/89 (99.0/1.0%)	637/6 (99.1/0.9%)	0.933
Apathy (No/Mild/Moderate-severe)	8896/241/69 (96.6/2.6/0.7%)	595/36/12 (92.5/5.6/1.9%)	** < 0.001**
Night-time behaviours (No/Mild/Moderate-severe)	8315/562/267 (90.9/6.1/2.9%)	569/46/27 (88.6/7.2/4.2%)	0.098
Appetite disorders (No/Mild/Moderate-severe)	8800/286/114 (95.7/3.1/1.2%)	586/40/17 (91.1/6.2/2.6%)	** < 0.001**

Bold denotes statistically significant differences between the two groups

### NPS and incident AD in CU individuals

**Fig. 2:**
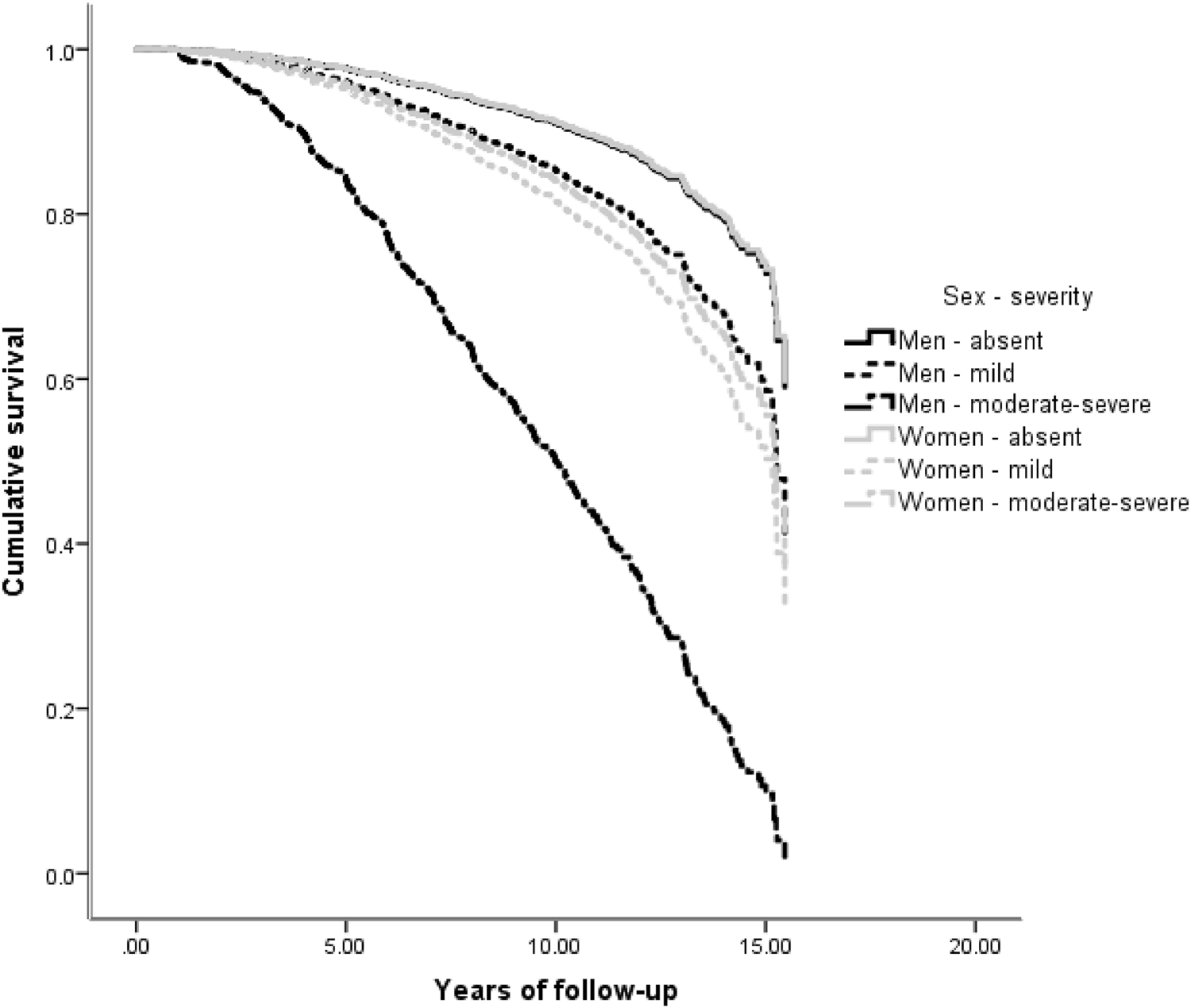
Heterogeneous associations between apathy and incident AD in cognitively unimpaired men and women

**Fig. 3:**
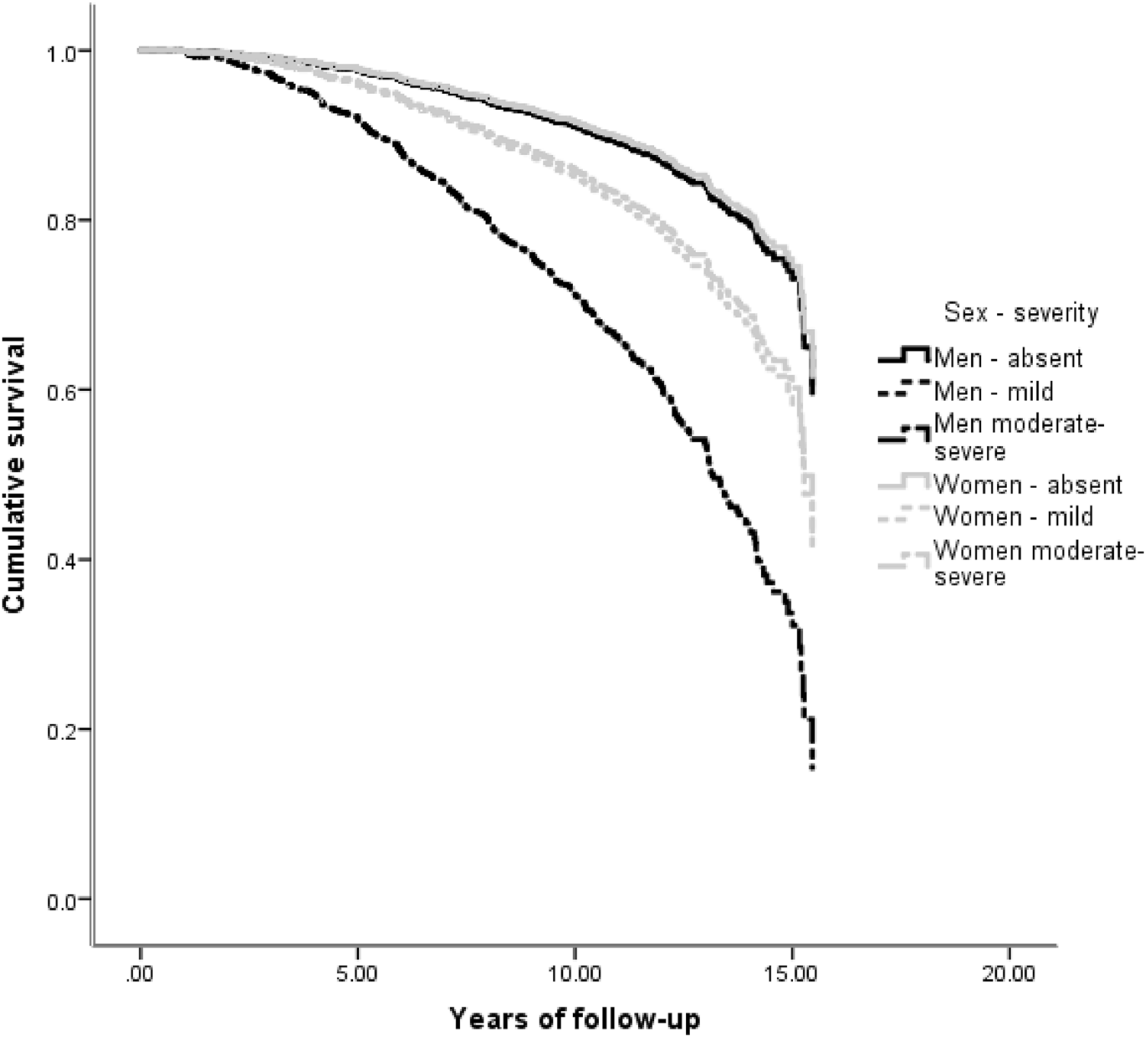
Heterogeneous associations between depression and incident AD in cognitively unimpaired men and women

**Fig. 4:**
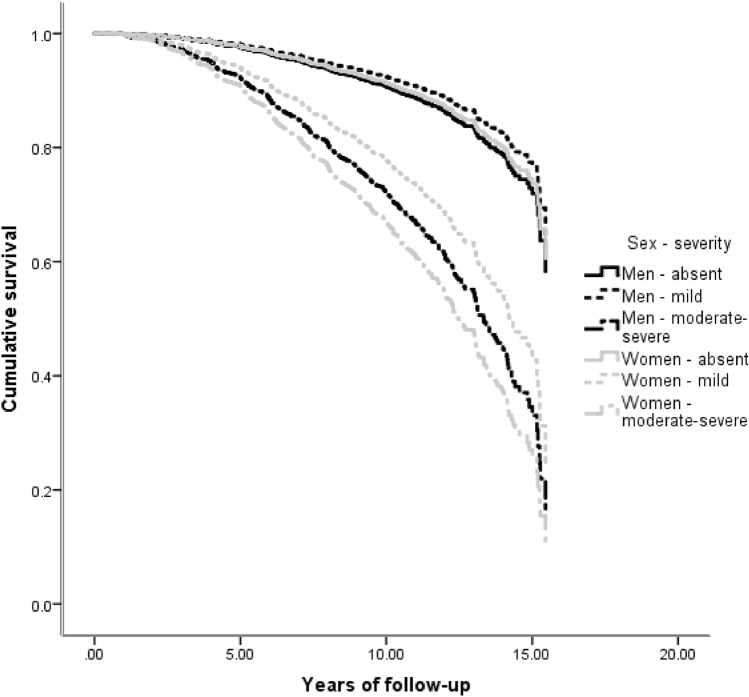
Heterogeneous associations between agitation and incident AD in cognitively unimpaired men and women

While many NPS were associated with an increased hazard of AD, the main effect of sex was not significant in any of the 12 survival analyses (Table 2, main effects). In most NPI-Q domains, NPS were comparably associated with future AD in men and women (Table 2, main effects): e.g., mild irritability was linked a two-fold hazard (HR = 2.01) of incident AD, while moderate-severe irritability was related to almost four-fold hazard (HR = 3.89) in both sexes. However, in the case of apathy, depression and agitation there were significant sex by NPS interactions. Moderate/severe apathy was linked to 7.36 greater overall risk of AD, but female sex substantially moderated this effect: women with moderate/severe apathy had one quarter (effect size = 0.26) of the aforementioned hazard (Fig. 2). Moderate-severe depression was related to 3.61 greater risk of progression to AD. Women with moderate/severe depression had only half (effect size = 0.48) this hazard. On the other hand, mild depression conferred an increased risk of AD only in women (female sex augmented the hazard by 83%) (Fig. 3). As for moderate-severe agitation, it was linked to an elevated hazard of progression to AD in both sexes (HR = 3.36), whereas mild symptoms conferred a greater hazard of AD only in women (243% greater than men) (Fig. 4). Confirmatory analyses accounting for global cognitive status practically reproduced the aforementioned findings, suggesting that the prognostic properties of the aforementioned NPS are independent of global cognition (Supplementary Table 1).

**Table 2:** Associations between neuropsychiatric symptoms and incident Alzheimer’s disease in cognitively unimpaired individuals

	Variable	Hazard ratio	Lower 95% CI	Upper 95% CI	*p*-value
Delusions	Absent	Ref			
	Present	3.53	00.87	140.3	0.077
	Men	Ref			
	Women	0.95	00.80	10.13	0.569
	Absent*sex	Ref			
	Present*sex	1.32	00.26	60.71	0.741
Hallucinations	Absent	Ref			
	Present	NA	00.00	10.06E + 178	0.968
	Men	Ref			
	Women	0.96	00.80	10.14	0.615
	Absent*sex	Ref			
	Present*sex	NA	00.00	50.08E + 185	0.966
Anxiety	Absent	Ref			0.229
	Mild disorder	1.29	00.75	20.22	0.364
	Moderate-severe disorder	1.79	00.84	30.81	0.133
	Men	Ref			
	Women	0.94	00.78	10.13	0.515
	Absent*sex	Ref			0.563
	Mild disorder*sex	1.40	00.73	20.68	0.309
	Moderate-severe disorder*sex	0.86	00.31	20.36	0.770
Depression	Absent	Ref			** < 0.001**
	Mild disorder	1.00	00.60	10.68	0.994
	Moderate-severe disorder	**3.61**	**20.08**	**60.28**	** < 0.001**
	Men	Ref			
	Women	0.94	00.78	10.13	0.498
	Absent*sex	Ref			**0.014**
	Mild disorder*sex	**1.83**	**10.02**	**30.30**	**0.043**
	Moderate-severe disorder*sex	0.48	00.23	10.01	0.053
Agitation	Absent	Ref			**0.003**
	Mild disorder	0.81	00.40	10.64	0.559
	Moderate-severe disorder	**3.36**	**10.65**	**60.86**	**0.001**
	Men	Ref			
	Women	0.93	00.78	10.11	0.420
	Absent*sex	Ref			**0.012**
	Mild disorder*sex	**3.43**	**10.51**	**70.79**	**0.003**
	Moderate-severe disorder*sex	1.33	00.49	30.60	0.580
Disinhibition	Absent	Ref			**0.028**
	Mild disorder	1.53	00.49	40.80	0.467
	Moderate-severe disorder	**3.30**	**10.34**	**80.12**	**0.010**
	Men	Ref			
	Women	0.96	00.80	10.15	0.646
	Absent*sex	Ref			0.602
	Mild disorder*sex	1.77	00.48	60.44	0.389
	Moderate-severe disorder*sex	0.70	00.18	20.70	0.609
Irritability	Absent	Ref			** < 0.001**
	Mild disorder	**2.01**	**10.36**	**20.98**	** < 0.001**
	Moderate-severe disorder	**3.89**	**20.35**	**60.44**	** < 0.001**
	Men	Ref			
	Women	1.06	00.88	10.29	0.524
	Absent*sex	Ref			0.222
	Mild disorder*sex	0.62	00.36	10.08	0.091
	Moderate-severe disorder*sex	0.81	00.38	10.75	0.593
Motor disorders	Absent	Ref			
	Present	0.37	00.05	20.66	0.323
	Men	Ref			
	Women	0.94	00.79	10.13	0.521
	Absent*sex	Ref			
	Present*sex	4.92	00.57	420.6	0.148
Elation	Absent	Ref			
	Present	2.38	00.76	70.47	0.137
	Men	Ref			
	Women	0.96	00.81	10.15	0.670
	Absent*sex	Ref			
	Present*sex	0.62	00.14	20.83	0.540
Apathy	Absent	Ref			**0.001**
	Mild disorder	1.69	00.96	20.97	00.70
	Moderate-severe disorder	**7.36**	**30.25**	**160.6**	** < 0.001**
	Men	Ref			
	Women	0.98	00.82	10.17	0.810
	Absent*sex	Ref			**0.048**
	Mild disorder*sex	1.31	00.64	20.67	0.455
	Moderate-severe disorder*sex	**0.26**	**00.08**	**00.81**	**0.020**
Night-time behaviours	Absent	Ref			0.124
	Mild disorder	1.12	00.62	20.00	0.716
	Moderate-severe disorder	1.84	10.02	30.30	0.042
	Men	Ref			
	Women	0.94	00.79	10.13	0.532
	Absent*sex	Ref			0.593
	Mild disorder*sex	1.43	00.72	20.83	0.307
	Moderate-severe disorder*sex	1.01	00.46	20.21	0.977
Appetite disorders	Absent	Ref			** < 0.001**
	Mild disorder	**2.26**	**10.33**	**30.85**	**0.003**
	Moderate-severe disorder	**5.01**	**20.03**	**120.4**	** < 0.001**
	Men	Ref			
	Women	0.97	00.81	10.17	0.769
	Absent*sex	Ref			0.738
	Mild disorder*sex	0.98	00.50	10.91	0.946
	Moderate-severe disorder*sex	0.65	00.22	10.91	0.436

*NA* non-applicable

Bold denotes statistically significant differences: between group differences were considered significant only if among group differences were determined significant, as well (*p*-value corresponding to the reference category of trichotomous variables); first the main effects of sex (male sex was used as the reference category) and NPS (absence of the respective NPS was used as the reference category) are provided and then sex by NPS interactions are quoted (male sex by NPS interactions was used as the reference category)

### MCI participant characteristics and missing data

Of the 44,713 participants of the UDS, a total of 6369 with MCI were eligible for the present analysis (Fig. 1). A total of 1773 were not included in any analysis due to missing data on covariates. An additional 151 were not involved in any model because of missing data on all NPS. Among the remaining 4445 participants, 0–4 individuals were excluded from each survival analysis (due to missing data on the specifically analysed NPS), with the exception of night-time behaviours (21 cases with missing data were excluded).

Individuals with missing data (n = 1,924) were older (76.8 ± 8.4 vs. 75.6 ± 8.1 years), had fewer apoE4 alleles, and were more often women, African American or Asian compared to those without missing data (who were more often men and white). DM, hypertension, dyslipidaemia, alcohol or other substance abuse, were more prevalent among those with missing data (missing data analysis not shown).

Throughout the average follow-up of 3.8 ± 3.0 years (range 0.4–15.5 years), 1467 older adults with MCI progressed to AD while 2978 did not. Baseline differences between those who did and did not develop AD are in Table 3.

**Table 3:** Baseline differences between individuals with mild cognitive impairment (MCI) who did and did not develop Alzheimer’s disease (AD) at follow-up

Variable	Non-demented at follow-up (*n* = 9211)	AD at follow-up (*n* = 643)	*p*-value
Age in years	74.6 ± 8.0	77.7 ± 7.9	** < 0.001**
Formal education in years	15.4 ± 3.3	15.5 ± 3.2	0.405
Sex (male/female)	1393/1585 (46.8/53.2%)	672/795 (45.8/54.2%)	0.543
Race (Caucasian/African American/American Indian or Alaska Native/Native Hawaiian or Pacific Islander/Asian/Multiracial)	2319/463/9/2/83/102 (77.9/15.5/0.3/0.1/2.8/3.4%)	1247/148/2/1/31/38 (85.0/10.1/0.1/0.1/2.1/2.6%)	** < 0.001**
Number of ApoE4 alleles (0/1/2)	1937/913/128 (65.0/30.7/4.3%)	756/568/143 (51.5/38.7/9.7%)	** < 0.001**
Parkinson’s disease (No/Yes)	2893/85 (97.1/2.9%)	1460/7 (99.5/0.5%)	** < 0.001**
Traumatic brain injury (No/Yes)	2573/405 (86.4/13.6%)	1310/157 (89.3/10.7%)	**0.006**
History of seizures (No/Yes)	2896/71 (97.6/2.4%)	1440/25 (98.3/1.7%)	0.140
B12 deficiency	2799/179 (94.0/6.0%)	1377/90 (93.9/6.1%)	0.870
Alcohol abuse (No/Yes)	2846/132 (95.6/4.4%)	1416/51 (96.5/3.5%)	0.131
Other substance abuse (No/Yes)	2939/39 (98.7/1.3%)	1463/4 (99.7/0.3%)	**0.001**
Smoking history (No/Yes)	1569/1379 (53.2/46.8%)	798/685 (54.8/45.2%)	0.321
Cardiovascular disease (No/Yes)	2499/479 (83.9/16.1%)	1259/208 (85.8/14.2%)	0.098
Cerebrovascular disease (No/Yes)	2652/323 (89.1/10.9%)	1315/151 (89.7/10.3%)	0.572
Diabetes Mellitus (No/Yes)	2477/494 (83.4/16.6%)	1253/213 (85.5/14.5%)	0.072
Hypertension (No/Yes)	1265/1710 (42.5/57.5%)	618/843 (42.3/57.7%)	0.889
Dyslipidaemia (No/Yes)	1090/1866 (36.9/63.1%)	580/878 (39.8/60.2%)	0.061
Antidepressants (No/Yes)	2289/689 (76.9/23.1%)	1161/306 (79.1/20.9%)	0.087
Antipsychotics (No/Yes)	2945/33 (98.9/1.1%)	1451/16 (98.9/1.1%)	0.958
Anxiolytics/sedatives/hypnotics (No/Yes)	2597/381 (87.2/12.8%)	1343/124 (91.5/8.5%)	** < 0.001**
Delusions (No/Yes)	2908/70 (97.6/2.4%)	1425/42 (97.1/2.9%)	0.305
Hallucinations (No/Yes)	2951/26 (99.1/0.9%)	1453/14 (99.0/1.0/ %)	0.788
Depression (No/Mild/Moderate-severe)	2257/494/223 (75.9/16.6/7.5%)	1070/297/100 (72.9/20.2/6.8%)	**0.011**
Anxiety (No/Mild/Moderate-severe)	2397/382/198 (80.5/12.8/6.7%)	1135/215/117 (77.4/14.7/8.0%)	**0.048**
Agitation (No/Mild/Moderate-severe)	2606/262/109 (87.5/8.8/3.7%)	1226/158/83 (83.6/10.8/5.7%)	**0.001**
Disinhibition (No/Mild/Moderate-severe)	2790/130/58 (93.7/4.4/1.9%)	1351/77/39 (92.1/5.2/2.7%)	0.123
Irritability (No/Mild/Moderate-severe)	2270/515/189 (76.3/17.3/6.4%)	1088/270/109 (74.2/18.4/7.4%)	0.228
Elation (No/Yes)	2934/44 (98.5/1.5%)	1446/21 (98.6/1.4%)	0.904
Motor symptoms (No/Yes)	2886/91 (96.9/3.1%)	1402/66 (95.5/4.5%)	**0.014**
Apathy (No/Mild/Moderate-severe)	2600/277/99 (87.4/9.3/3.3%)	1218/187/62 (83.0/12.7/4.2%)	** < 0.001**
Night-time behaviours (No/Mild/Moderate-severe)	2392/356/212 (80.8/12.0/7.2%)	1190/181/93 (81.3/12.4/6.4%)	0.592
Appetite disorders (No/Mild/Moderate-severe)	2681/220/74 (90.1/7.4/2.5%)	1289/129/48 (87.9/8.8/3.3%)	0.074

Bold denotes statistically significant differences between the two groups

### NPS and incident AD in men and women with MCI

NPS were similarly associated with incident AD in men and women (Table 4). Moderate/severe apathy, in specific, was related to ~ 1.90 greater hazard of progressing to AD in both sexes; therefore, its hazard conferring properties in men were substantially attenuated compared to the CU set. In general, NPS conferred a lesser hazard to individuals with MCI in comparison with CU people, as reflected on the effect size of the associations. Confirmatory analyses accounting for global cognition reproduced these findings (Supplementary Table 2).

**Table 4:** Associations between neuropsychiatric symptoms and incident Alzheimer’s disease in individuals with mild cognitive impairment

	Variable	Hazard ratio	Lower 95% CI	Upper 95% CI	*p*-value
Delusions	Absent	Ref			
	Present	1.57	00.97	20.56	0.069
	Men	Ref			
	Women	1.05	00.94	10.17	0.364
	Absent*sex	Ref			
	Present*sex	0.97	00.52	10.81	0.913
Hallucinations	Absent	Ref			
	Present	**2.12**	**10.17**	**30.86**	**0.014**
	Men	Ref			
	Women	1.06	00.95	10.18	0.275
	Absent*sex	Ref			
	Present*sex	0.38	00.11	10.37	0.138
Anxiety	Absent	Ref			**0.001**
	Mild disorder	**1.39**	**10.11**	**10.73**	**0.004**
	Moderate-severe disorder	**1.48**	**10.12**	**10.97**	**0.007**
	Men	Ref			
	Women	1.06	00.94	10.20	0.349
	Absent*sex	Ref			0.840
	Mild disorder*sex	0.92	00.69	10.24	0.583
	Moderate-severe disorder*sex	1.03	00.70	10.51	0.879
Depression	Absent	Ref			0.075
	Mild disorder	1.17	00.96	10.44	0.122
	Moderate-severe disorder	1.34	00.99	10.82	0.063
	Men	Ref			
	Women	1.02	00.90	10.15	0.793
	Absent*sex	Ref			0.056
	Mild disorder*sex	1.27	00.98	10.65	0.075
	Moderate-severe disorder*sex	0.75	00.50	10.14	0.173
Agitation	Absent	Ref			** < 0.001**
	Mild disorder	**1.48**	**10.17**	**10.88**	**0.001**
	Moderate-severe disorder	**1.93**	**10.41**	**20.63**	** < 0.001**
	Men	Ref			
	Women	1.07	00.95	10.20	0.265
	Absent*sex	Ref			0.676
	Mild disorder*sex	1.06	00.76	10.47	0.754
	Moderate-severe disorder*sex	0.83	00.53	10.31	0.426
Disinhibition	Absent	Ref			**0.007**
	Mild disorder	**1.52**	**10.09**	**20.12**	**0.015**
	Moderate-severe disorder	**1.63**	**10.04**	**20.54**	**0.033**
	Men	Ref			
	Women	1.05	00.94	10.17	0.421
	Absent*sex	Ref			0.532
	Mild disorder*sex	1.23	00.77	10.95	0.385
	Moderate-severe disorder*sex	1.28	00.67	20.43	0.453
Irritability	Absent	Ref			**0.008**
	Mild disorder	1.17	00.97	10.42	0.103
	Moderate-severe disorder	**1.47**	**10.13**	**10.90**	**0.004**
	Men	Ref			
	Women	1.04	00.92	10.18	0.519
	Absent*sex	Ref			0.284
	Mild disorder*sex	1.23	00.94	10.61	0.132
	Moderate-severe disorder*sex	0.94	00.63	10.41	0.766
Motor disorders	Absent	Ref			
	Present	**1.69**	**10.19**	**20.42**	**0.004**
	Men	Ref			
	Women	1.05	00.94	10.17	0.414
	Absent*sex	Ref			
	Present*sex	1.49	00.91	20.44	0.117
Elation	Absent	Ref			
	Present	1.21	00.66	20.20	0.542
	Men	Ref			
	Women	1.05	00.94	10.17	0.374
	Absent*sex	Ref			
	Present*sex	1.28	00.54	30.04	0.577
Apathy	Absent	Ref			** < 0.001**
	Mild disorder	**1.78**	**10.43**	**20.22**	** < 0.001**
	Moderate-severe disorder	**1.90**	**10.34**	**20.70**	** < 0.001**
	Men	Ref			
	Women	1.09	00.97	10.22	0.166
	Absent*sex	Ref			0.786
	Mild disorder*sex	0.90	00.66	10.22	0.487
	Moderate-severe disorder*sex	0.99	00.59	10.66	0.962
Night-time behaviours	Absent	Ref			0.375
	Mild disorder	1.17	00.94	10.47	0.165
	Moderate-severe disorder	1.06	00.77	10.44	0.731
	Men	Ref			
	Women	1.04	00.93	10.17	0.476
	Absent*sex	Ref			0.811
	Mild disorder*sex	1.06	00.77	10.45	0.726
	Moderate-severe disorder*sex	1.13	00.74	10.73	0.564
Appetite disorders	Absent	Ref			**0.025**
	Mild disorder	**1.44**	**10.07**	**10.92**	**0.016**
	Moderate-severe disorder	1.37	00.87	20.14	0.170
	Men	Ref			
	Women	1.03	00.92	10.15	0.609
	Absent*sex	Ref			0.636
	Mild disorder*sex	1.19	00.82	10.73	0.358
	Moderate-severe disorder*sex	1.09	00.61	10.96	0.769

*NA* non-applicable

Bold denotes statistically significant differences: between group differences were considered significant only if among group differences were determined significant, as well (*p*-value corresponding to the reference category of trichotomous variables); first the main effects of sex (male sex was used as the reference category) and NPS (absence of the respective NPS was used as the reference category) are provided and then sex by NPS interactions are quoted (male sex by NPS interactions was used as the reference category)

## Discussion

The present study revealed that apathy, depression and agitation are differentially associated with incident AD in CU men and women. Moderate-severe apathy was the strongest predictor of future AD in CU men but conferred a lesser hazard of incident AD in CU women. Mild depression and agitation increased the hazard of conversion to AD in CU women but not men, while moderate to severe depression was linked to an elevated hazard of progression to AD in both sexes. However, moderate-severe depression was linked to a markedly elevated risk of future AD in CU men compared to women. On the other hand, NPS were similarly associated with future AD in men and women with MCI. Intriguingly, as with previous research, NPS conferred a greater risk to CU individuals rather than to people with MCI, as reflected on the effect size of the estimated associations. Of note, these estimates were underpowered in the investigation of psychotic symptoms (they had the lowest prevalence among all NPS, which is reflected on the precision of our estimates).

Apathy is conceptualized as the lack of motivation and goal-pursuing behaviours while emotional flattening and indifference usually coexist [32]. The experience of negative rather than blunted affect distinguishes depression from apathy [33]. These neuropsychiatric manifestations constitute the most common NPS in people with MCI and AD [1, 34]. Both depressive symptoms and apathy have been consistently associated with an increased risk of incident AD in populations with MCI or intact cognition [5, 11, 14, 35]. However, no published study has investigated sex interactions. Regarding apathy, our findings appear to be in line with its greater burden in men compared to women with AD [10]. Considering the low prevalence of apathy in the general, CU population, it is possible that the identification of moderate-severe symptoms may yield a relatively strong positive prognostic value in the detection of CU male individuals at high-risk of developing AD [36].

Moderate-severe depression was also a moderate to strong predictor of incident AD in CU men. Given, however, the higher prevalence of the disorder in older populations, moderate-severe depressive symptoms should raise physicians’ vigilance for future AD despite a lesser transition hazard than apathy in CU men [37, 38]. On the other hand, depression only conferred a low hazard for AD in women. Based on the well-established female preponderance of this highly-prevalent condition in later life, depressive symptoms may not be major predictors of AD in CU women [37, 38].

Conversely, delusions and hallucinations are very uncommon in the CU, general population and slightly more prevalent in individuals with MCI [39, 40]. It is argued that psychotic symptoms constitute the strongest NPS precursors of dementia, especially in CU older adults [5, 12]. Of note, delusions and hallucinations have stronger affinity to dementia with Lewy bodies, frontotemporal and vascular dementia, compared to AD [5]. Therefore, despite their well-established low sensitivity for AD, late-life onset psychosis is likely a harbinger of all-cause dementia. Close surveillance is warranted when late-life delusions or hallucinations present in either men or women.

Lability symptoms (agitation, irritability, disinhibition, elation, motor disturbances) and appetite disorders, on the other hand, are much more common than psychosis in individuals with intact cognition or MCI [39] and account for a significant portion of caregiver burden [41]. The relationship between these manifestations and incident dementia is less prominent with the published literature suggesting a predominant link to later frontotemporal dementia, and to a lesser extent AD [5, 35]. Apart from mild agitation, this association with AD is sex-independent, and the presence of lability symptoms and appetite disorders should alert physicians to a greater hazard of future AD in both men and women with intact cognition or MCI.

As for sleep disturbances, there is a vast literature confirming their association with future AD, using more thorough assessment protocols than the NPI-Q, separately and meticulously addressing the different aspects of sleep [42]. Of note, sleep disorders are associated with future risk of all-cause dementia and cognitive decline without specific sex-dependent associations known to date.

### Strengths and limitations

The main strengths of our study are the large sample size, the long follow-up and the large number of documented events (incident AD). We were careful to exclude individuals with pre-existing psychiatric disorders that may interfere with cognition, as well as to account for important confounders.

This analysis has several weaknesses, as well. First, the diagnosis of AD and other dementias was established by either the examining physician or by an expert-consensus team, based on comprehensive neurological and neuropsychological evaluations (imaging and biological biomarkers were not uniformly available). Although, the exhaustive assessments of the UDS improve the accurate diagnostic characterization of the participants, the presence of misclassification bias cannot be ruled out, especially for cases of mixed dementia. Second, the prevalence of psychosis was low, especially among CU individuals. Therefore, some analyses were underpowered. Third, NPS were assessed using the NPI-Q: while this is a widely used instrument in dementia research, more rigorous assessment tools (e.g., NPI-C- clinician inventory) would be more sensitive in revealing, and more accurate in quantifying, NPS severity. Therefore, more thorough assessment protocols might capture additional associations. Moreover, although we adjusted analyses for several factors, our findings may have been driven by residual confounding (it would not be possible to capture the effect of every potential confounder [43, 44]) or the non-trivial proportion of missing data. Another limitation of this study is its observational nature. In specific, it is not possible to make etiologic inferences about NPS and incident AD, considering that preclinical neurodegenerative brain changes precede the identification of AD for many years. Finally, the current report focused exclusively on AD; therefore, future research ought to investigate sex differences in other dementia entities, as well.

## Conclusions

We found that apathy, depression, and agitation are differentially associated with incident AD in CU men versus women. No NPS were related to different risks of progression to AD in older adults with MCI. Of interest, as with previous research, NPS conferred a greater risk to CU individuals rather than to people with MCI, as reflected on the effect size of the associations. These findings may have implications in the early identification of people at high risk to develop AD.

## Supplementary Information

Below is the link to the electronic supplementary material.

Supplementary file1 (DOCX 26 KB)

Supplementary file2 (DOCX 26 KB)

## Data Availability

For further information on access to the NACC database, please contact NACC (contact details can be found at https://naccdata.org/).
