# Supplementary material for: Associations between neuropsychiatric symptoms and incident Alzheimer’s dementia in men versus women
Source: J Neurol. Author manuscript; Available in PMC 2023 Apr 1. (PMC10025238; doi:10.1007/s00415-022-11541-w)
Supplement: 1866856_Sup_Tab_2 [file NIHMS1866856-supplement-1866856_Sup_Tab_2.docx]

**Supplementary Table 2** Associations between neuropsychiatric symptoms and incident Alzheimer’s disease in individuals with mild cognitive impairment after adjusting for global cognitive scores at baseline.

|  | **Variable** | **Hazard ratio** | **Lower 95%CI** | **Upper 95%CI** | **p-value** |
| --- | --- | --- | --- | --- | --- |
| Delusions | Absent | Ref | | |  |
|  | Present | 1.32 | 0.80 | 2.19 | .277 |
|  | Men | Ref | | |  |
|  | Women | **1.16** | **1.04** | **1.29** | **.009** |
|  | Absent*sex | Ref | | |  |
|  | Present*sex | 0.99 | 0.51 | 1.90 | .970 |
| Hallucinations | Absent | Ref | | |  |
|  | Present | **3.04** | **1.67** | **5.54** | **< .001** |
|  | Men | Ref | | |  |
|  | Women | **1.17** | **1.05** | **1.31** | **.005** |
|  | Absent*sex | Ref | | |  |
|  | Present*sex | 0.29 | 0.08 | 1.04 | .058 |
| Anxiety | Absent | Ref | | | **.004** |
|  | Mild disorder | **1.36** | **1.09** | **1.70** | **.007** |
|  | Moderate-severe disorder | **1.41** | **1.06** | **1.88** | **.018** |
|  | Men | Ref | | |  |
|  | Women | **1.16** | **1.03** | **1.31** | **.018** |
|  | Absent*sex | Ref | | | .985 |
|  | Mild disorder*sex | 0.98 | 0.73 | 1.32 | .897 |
|  | Moderate-severe disorder*sex | 0.97 | 0.66 | 1.43 | .893 |
| Depression | Absent | Ref | | | .123 |
|  | Mild disorder | 1.09 | 0.89 | 1.34 | .409 |
|  | Moderate-severe disorder | 1.36 | 1.00 | 1.86 | .049 |
|  | Men | Ref | | |  |
|  | Women | 1.12 | 0.98 | 1.27 | .087 |
|  | Absent*sex | Ref | | | **.015** |
|  | Mild disorder*sex | **1.33** | **1.02** | **1.74** | **.035** |
|  | Moderate-severe disorder*sex | 0.70 | 0.46 | 1.06 | .094 |
| Agitation | Absent | Ref | | | **< .001** |
|  | Mild disorder | **1.48** | **1.16** | **1.89** | **.002** |
|  | Moderate-severe disorder | **1.93** | **1.41** | **2.63** | **< .001** |
|  | Men | Ref | | |  |
|  | Women | **1.19** | **1.05** | **1.34** | **.005** |
|  | Absent*sex | Ref | | | .566 |
|  | Mild disorder*sex | 0.98 | 0.70 | 1.38 | .919 |
|  | Moderate-severe disorder*sex | 0.78 | 0.50 | 1.23 | .286 |
| Disinhibition | Absent | Ref | | | **< .001** |
|  | Mild disorder | **1.84** | **1.31** | **2.57** | **< .001** |
|  | Moderate-severe disorder | **1.59** | **1.01** | **2.48** | **.043** |
|  | Men | Ref | | |  |
|  | Women | **1.16** | **1.03** | **1.30** | **.012** |
|  | Absent*sex | Ref | | | .567 |
|  | Mild disorder*sex | 1.02 | 0.64 | 1.62 | .934 |
|  | Moderate-severe disorder*sex | 1.42 | 0.75 | 2.69 | .287 |
| Irritability | Absent | Ref | | | **.001** |
|  | Mild disorder | 1.20 | 0.99 | 1.45 | .068 |
|  | Moderate-severe disorder | **1.61** | **1.24** | **2.09** | **< .001** |
|  | Men | Ref | | |  |
|  | Women | 1.17 | 1.03 | 1.33 | .014 |
|  | Absent*sex | Ref | | | .239 |
|  | Mild disorder*sex | 1.16 | 0.89 | 1.52 | .282 |
|  | Moderate-severe disorder*sex | 0.79 | 0.53 | 1.18 | .250 |
| Motor disorders | Absent | Ref | | |  |
|  | Present | 1.38 | 0.96 | 1.98 | .078 |
|  | Men | Ref | | |  |
|  | Women | **1.15** | **1.03** | **1.28** | **.015** |
|  | Absent*sex | Ref | | |  |
|  | Present*sex | 1.62 | 0.97 | 2.68 | .063 |
| Elation | Absent | Ref | | |  |
|  | Present | 1.06 | 0.58 | 1.93 | .856 |
|  | Men | Ref | | |  |
|  | Women | **1.16** | **1.04** | **1.29** | **.010** |
|  | Absent*sex | Ref | | |  |
|  | Present*sex | 1.16 | 0.49 | 2.77 | .736 |
| Apathy | Absent | Ref | | | **< .001** |
|  | Mild disorder | **1.60** | **1.28** | **2.00** | **< .001** |
|  | Moderate-severe disorder | **1.77** | **1.24** | **2.51** | **.002** |
|  | Men | Ref | | |  |
|  | Women | **1.19** | **1.05** | **1.34** | **.005** |
|  | Absent*sex | Ref | | | .816 |
|  | Mild disorder*sex | 0.92 | 0.67 | 1.26 | .595 |
|  | Moderate-severe disorder*sex | 1.09 | 0.65 | 1.82 | .756 |
| Night-time behaviours | Absent | Ref | | | .115 |
|  | Mild disorder | 1.27 | 1.01 | 1.59 | .038 |
|  | Moderate-severe disorder | 1.06 | 0.77 | 1.44 | .736 |
|  | Men | Ref | | |  |
|  | Women | **1.16** | **1.03** | **1.31** | **.015** |
|  | Absent*sex | Ref | | | .892 |
|  | Mild disorder*sex | 0.98 | 0.72 | 1.35 | .920 |
|  | Moderate-severe disorder*sex | 1.11 | 0.72 | 1.70 | .649 |
| Appetite disorders | Absent | Ref | | | .888 |
|  | Mild disorder | 1.15 | 0.61 | 2.16 | .672 |
|  | Moderate-severe disorder | 1.14 | 0.43 | 3.03 | .791 |
|  | Men | Ref | | |  |
|  | Women | **1.14** | **1.01** | **1.27** | **.032** |
|  | Absent*sex | Ref | | | .628 |
|  | Mild disorder*sex | 1.18 | 0.81 | 1.72 | .392 |
|  | Moderate-severe disorder*sex | 1.16 | 0.64 | 2.10 | .623 |

**Bold** denotes statistically significant differences: between group differences were considered significant only if among group differences were determined significant, as well (p-value corresponding to the reference category of trichotomous variables); first the main effects of sex (male sex was used as the reference category) and NPS (absence of the respective NPS was used as the reference category) are provided and then sex by NPS interactions are quoted (male sex by NPS interactions was used as the reference category).
